# Effect of Long-Term Thermal Aging on Microstructure Evolution and Creep Deformation Behavior of a Novel 11Cr-3W-3Co Martensite Ferritic Steel

**DOI:** 10.3390/ma15103659

**Published:** 2022-05-20

**Authors:** Hongchang Zhao, Xi Han, Mingjia Wang, Zixi Wang

**Affiliations:** 1 Key Laboratory of Metastable Materials Science and Technology, College of Materials Science and Engineering, Yanshan University, Qinhuangdao 066004, China; hczhaoysu@163.com; 2 Department of Chemical Engineering and Modern Material, Shangluo University, Shangluo 726000, China; xihan202203@163.com; 3 CITIC Dicastal Co., Ltd., Qinhuangdao 066011, China

**Keywords:** aging, precipitated phase, coarsening behavior, subgrain, creep deformation

## Abstract

This paper focused on the microstructure evolution under different thermal aging times at 650 °C and its effect on creep behavior in 11Cr-3W-3Co heat-resistant steel. After short-term thermal aging at 650 °C (>750 h), a Laves phase was found in the regions adjacent to the PAG boundaries, martensitic lath boundaries, and M23C6 carbides, and gradually swallowed adjacent M23C6 carbides with the aging time increased. Higher contents of Si and P are good promoters of the nucleation of the Laves phase during long-term aging. In addition, the coarsening behavior of the Laves phase, M23C6, and MX were investigated. As the aging time increases, the coarsening behavior among precipitated phases in the above-mentioned example exhibits remarkable variability, which is discussed in detail in this paper, and the evolution of the subgrain size was also analyzed in detail. The increasing rate of subgrain size is, in general, consistent with that of the M23C6 carbide size. The evolution of dislocation density in different aging times shows an obvious difference, and the decreasing rate of dislocation density is significantly affected by the precipitated phase after long-term aging time. The creep performance of the material decreases significantly as the aging time increases, which is closely related to the coarsening of the precipitates such as M23C6 carbides and subgrain during long-term aging.

## 1. Introduction

Ultra-supercritical technology is a highly efficient thermal power generation technology that is being developed around the world. The steam temperature of ultra-supercritical units is gradually increased based on the urgent requirement to obtain higher thermal efficiency and less energy consumption, which implies that the requirements for high-temperature microstructural stability and high-temperature mechanical properties of materials are further enhanced [1,2,3,4,5].

11Cr-3W-3Co steel is designed and optimized by adding Co, W, V, Nb, N, B, and other elements based on the traditional 9–12% Cr martensitic heat-resistant steel, where the addition of 3.0 wt.% of Co and W further improves the creep strength by solid solution strengthening compared with other steels of the same grade [6], and the addition of small amounts of B inhibits the coarsening of carbides and increases the creep strength by stabilizing the subgrain boundary. Therefore, it is widely used in the manufacturing of large diameter and heavy wall thickness pipes, turbine blades, bolts, and other critical components in ultra-supercritical units for up to 630 °C on the basis of its excellent creep strength, good oxidation resistance, and corrosion resistance.

The structural reliability of materials in components serving in high temperature and stress environments strongly depends on creep strength [7,8,9,10]. Creep strength is mainly determined by the stability of microstructures such as martensitic laths and precipitates in steel [11,12]. Maruyama et al. [13] reported that M23C6 precipitates play a more important role in controlling subgrain coarsening during long-term creep compared with MX precipitates due to higher volume fraction, and the average size of the M23C6 particles is equivalent to that of the subcrystalline grains, which is a decisive factor in stabilizing subcrystalline boundaries [14]. Isik et al. [15] found that the Laves phase had a significant effect on both the microstructure and creep properties and was regarded as detrimental to creep strength. HORIUCHI et al. showed the size of M23C6 particles changes considerably during long-term aging or creep, and therefore has a significant effect on subgrain size, while MX size remains essentially constant, so the effect of aging on creep deformation is mainly caused by changes in M23C6 particle size [16,17]. Zhao et al. [18] revealed the microscopic mechanism of creep fracture 9Cr-3W-3Co heat-resistant steel under creep conditions at 650 °C. The excellent high-temperature mechanical properties of 9Cr-3W-3Co heat-resistant steel are mainly attributed to the solution strengthening caused by Co and W atoms and the high-density dislocations distributed in the matrix, while the precipitates are mainly pinned dislocations and hinder the movement of the dislocations.

Thus, the current article focuses on analyzing the nucleation and coarsening behavior among different precipitate phases and the evolution of subgrain in 11Cr-3W-3Co steel under different aging conditions at 650 °C. The creep deformation behavior under different aging conditions was discussed. Furthermore, detailed microstructural characteristics and fracture surfaces were also analyzed.

## 2. Materials and Methods

The test material selected for this study, 11Cr-3W-3Co, is martensitic ferritic heat-resistant steel with the chemical composition listed in Table 1. The chemical composition and elemental content of the test steel used in this experiment were measured by spectrometer (SPECTROMAXx, SPECTRO, Kleve, Germany). The heat-resistant steel was austenitized at 1100 °C for 1.5 h (oil cooling) and then tempered at 710 °C for 4 h (air cooling). Subsequently, thermal aging experiments were performed at 650 °C at different aging times shown in Table 2. Furthermore, creep tests were conducted at 650 °C/160 MPa for different aging samples by applying an electronic creep tester (RSW50, NCS, Shanghai, China).

Scanning electron microscope (S3400N, Hitachi, Tokyo, Japan) was employed for observation and analysis of martensitic laths and the size and distribution of precipitates, and further identification of the different phases according to the Z-contrast in backscattered electrons (BSE) images [19,20,21]. A transmission electron microscope (Talos-F200X, FEI Corporation, Hillsboro, OR, USA) was applied to analyze the laths’ morphology, dislocations, and precipitates; and to identify the composition of precipitates of different morphologies by energy-dispersive spectrum (EDS). The thin samples prepared for TEM observation were mechanically ground to the thickness of 50 µm and followed by electropolishing in a double-jet apparatus with a mixed solution of 10% perchloric acid and 90% glacial acetic acid at 10 °C.

In this paper, an X-ray diffractometer (D/max-2500PC, Rigaku, Tokyo, Japan) was employed to calculate and analyze the dislocation density in macroscopic areas within the test steel for different aging conditions. To ensure that the surface of the material is free of residual stresses, electrolytic polishing was performed using a 6% solution of perchloric acid in anhydrous ethanol. The voltage and the current were controlled at 10 V and 350 mA, respectively. The XRD patterns for different specimens were obtained by a slow scan conducted at a large angular interval from 20° to 140°, with a scan speed of 0.5°/min voltage~40 kV and current~200 mA.

## 3. Results and Discussion

### 3.1. Precipitation Behavior of Different Types of Precipitated Phases

**Figure 1:**
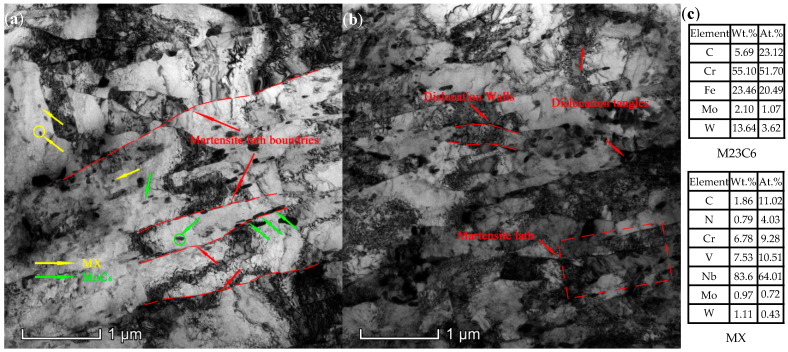
TEM image of the test steel in the original state: (**a**) Cr-rich (M23C6) carbides precipitated at martensite lath boundaries and Nb-rich (MX) precipitates in martensite laths; (**b**) typical tempered martensite laths with high dislocation density; dislocation walls were evolved from entangled dislocations; and (**c**) the chemical composition of the M23C6 carbide and Nb-rich (MX) carbonitrides.

As shown in Figure 1a, only two precipitation phases, M23C6 carbides and MX carbonitrides, were observed except for the Laves phase in its initial state. Furthermore, Cr-rich (M23C6) carbides were mainly located along the martensitic lath boundaries and prior austenite grain (PAG) boundaries, while finely dispersed Nb-rich (MX) carbonitrides tended to precipitate inside the laths. The typical tempered martensitic laths with a high density of dislocations are illustrated in Figure 1b, and the dislocation walls can also be clearly observed, which evolved from entangled dislocations.

**Figure 2:**
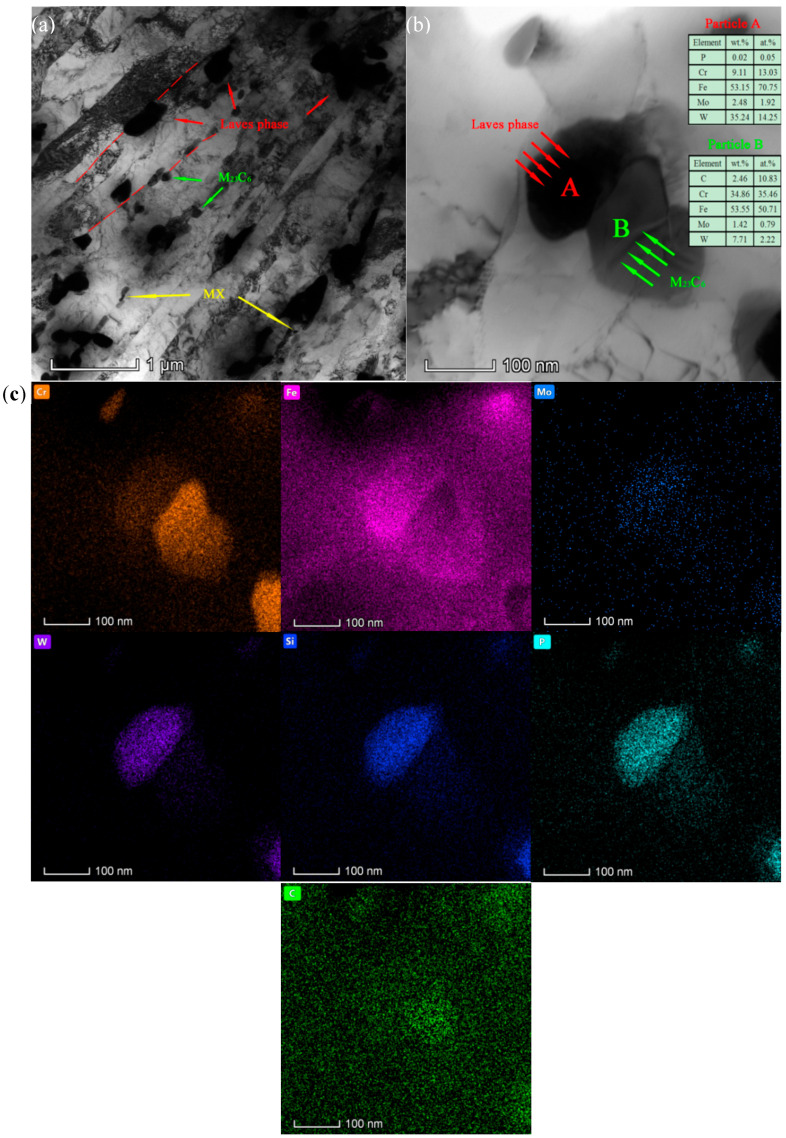
TEM and EDS images of the test steel aged at 650 °C: (**a**) TEM BF image, precipitation of different phases aged for 750 h; (**b**) TEM BF image—the engulfing process of Laves phase for 3000 h; and (**c**) surface distribution maps of the selected area in (**b**) for Cr, Fe, Mo, W, Si, P, and C.

Some irregularly-shaped Laves phases were observed to precipitate preferentially along the martensitic lath boundaries and PAG boundaries when the aging time reached 750 h as shown in Figure 2a. As the aging time increases to 3000 h, Figure 2b clearly illustrates that Laves phases form in the vicinity of the M23C6 particles and gradually swallow the adjacent Cr-rich M23C6 carbides, which is in accordance with the previous results [22]. Furthermore, the elemental mapping distributions originating from the EDS X-ray spectra (Figure 2c) reveal that the Laves phase is primarily composed of W, Mo, Fe, Cr, P, and Si, which is consistent with the results of earlier studies [15]. Xu et al. [21] investigated the nucleation mechanism and elemental composition and content of the Laves phase in 12% Cr heat-resistant steel in detail, and found the Laves phase mainly consists of W, Mo, Fe, Cr, Si, and P. Furthermore, two mechanisms of the nucleation were summarized and analyzed in detail. One is that the Laves phase form preferentially on the PAGBs and martensitic lath boundaries. Another is that the Laves phase tends to nucleate at the interface of M23C6 particles and subsequently grows obviously in size until it swallows all the M23C6 particles, which is in accordance with previous research results. It is well illustrated in Figure 2c that there are clear segregations of Si and P elements around the Laves phase. Hosoi et al. and Kato et al. [23,24] also confirmed that Si and P are the necessary elements for the nucleation of the Laves phase in 9–12% Cr steels, and the precipitation of the Laves phase is significantly retarded by the reduction of Si content in the steel. With regard to the factors affecting the formation of the Laves phase, previous results [25] have shown that the stability of the Laves phase is determined by the atomic size and the average electron concentration; Si element was found to reduce the average electron concentration in ternary alloys containing the Laves phase [26]. Thus, the nucleation and coarsening behavior of the Laves phase are strongly affected by the Si element. In addition, there is a relatively obvious tendency for carbon atoms to segregate in the vicinity to the phase interface between the Laves phase and the martensitic matrix from Figure 2c, which is consistent with the previous result [27,28]. The reason for this is that the decomposition of M23C6 and the continuous growth of the Laves phase can promote the release of C atoms. Moreover, phase boundaries and stacking fault around the phase boundaries were the most efficient sites for trapping C atoms where the diffusion rate along the dislocation cores is higher than the bulk diffusion of solid solution elements.

### 3.2. The Coarsening Behavior of Precipitated Phases

Statistical results of the coarsening kinetics for all precipitate particles respectively with different thermal aging times at 650 °C were obtained from a large number of TEM images, counting 80 particles for each precipitate at each given aging condition. The average sizes are determined by the arithmetic mean of all the particles sizes, the number densities are considered as the number of precipitate particles per unit area, and the area fractions are obtained by dividing the projected area of all the particles by the area of the TEM images used for the statistics.

**Figure 3:**
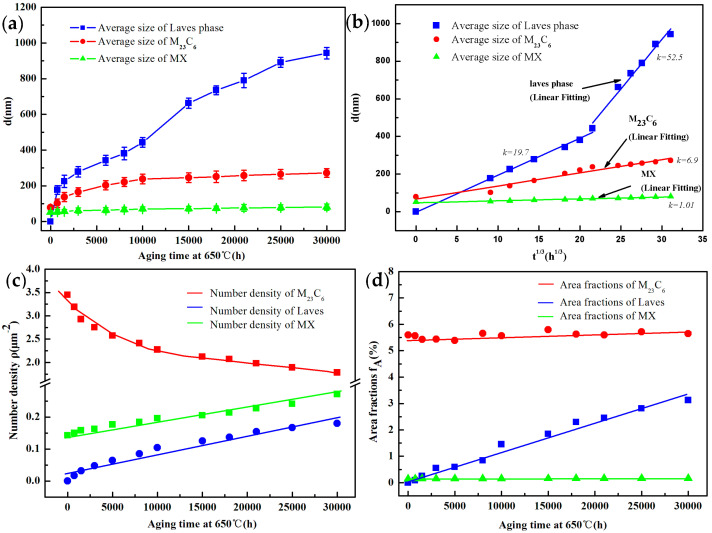
The coarsening behavior of precipitates (M23C6, Laves phase and MX) and evolution of subgrain width during long-term thermal aging: (**a**) average diameters; (**b**) d as a function of *t*^1/3^; (**c**) number densities; and (**d**) area fractions.

As shown in Figure 3a, the statistical results of the average size for the three types of precipitates illustrate that the MX carbide is fairly stable in size, increasing slightly from 53 nm to 81 nm after aging for 30,000 h. While the size of the Laves phase particles shows a remarkable increase once formed, which starts from 176 nm to 943 nm as the aging time increased to 30,000 h. Combined with the above statistic results of the average size, it obviously shows that the average diameter of the M23C6 particles increases steadily from the initial state to the aged stage for 30,000 h, and the tendency to enter a stabilization period does not occur.

Figure 3b illustrates the average size d as a function of *t*^1/3^ for the three different types of precipitated phases. The relationships between the average particle sizes and the thermal aging time of M23C6 and MX are essentially in line with the linear law. The coarsening characteristics of the precipitates conform to the coarsening kinetics formula of multi-component alloys, and the coarsening kinetics can be expressed by the following Formula (1) [29], which is in agreement with the Lifshitz–Slyozov–Wagner (LSW) model for Ostwald ripening during long-term thermal aging [14,30]:

(1) $${\bar{r}=kt}^{1/3}$$

where *k* is the coarsening rate, *t* is the thermal aging time, and $\bar{r}$ is the average size of the precipitated phase.

The coarsening rate *k* can be expressed by the linear slope in Figure 3b, with values of 6.9 and 1.01 nm/h^1/3^ corresponding to M23C6 and MX, respectively. However, the average diameter d as a function of *t*^1/3^ exhibits significant differences in different thermal aging periods for the Laves phase. The coarsening rates are 19.7 nm/h^1/3^ (<10,000 h) and 52.5 nm/h^1/3^ (>10,000 h), respectively.

Figure 3c illustrates the variations of the number density of M23C6, Laves phase, and MX with different aging times, respectively. The number density of M23C6 carbide is much higher than those of MX carbonitride and the Laves phase. A remarkable decrease in the number density of M23C6 can be seen from 3.45 to 1.78 mm^−2^ for up to 30,000 h, while the number densities of MX increase rather slowly from 0.14 to 0.21 mm^−2^. In contrast, the number density of the Laves phase maintains a small increase from 0.017 to 0.135 mm^−2^. Statistical results of the number density illustrate that the number density value of M23C6 is still much larger than that of the Laves phase and MX at 30,000 h aging time.

The volume fractions of M23C6 and MX remained stable during the long-term thermal aging process. Nevertheless, the Laves phase increased slightly from 0.25% after 1500 h to 3.13% after 30,000 h, as shown in Figure 3d.

Statistical results of the coarsening behavior showed that the size and number of MX carbides did not change significantly and remained in a relatively stable state during long-term aging. The evolution of the size of the M23C6 carbides fits the Ostwald ripening model, and the Laves phase particles do not reach thermodynamic equilibrium and still undergo significant changes in size and number, which is consistent with previous studies [31,32].

### 3.3. Evolution of Subgrain and Dislocation Density 

**Figure 4:**
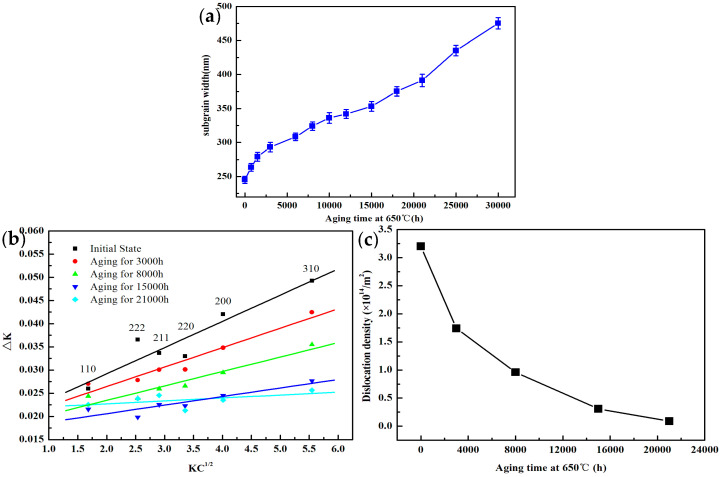
Evolution of subgrain size and calculation of dislocation density: (**a**) dislocation density as a function of aging time at 650 °C; (**b**) linear relationship between ΔK and $K\bar{C}$^1/2^ in modified Williamson–Hall equation; and (**c**) dislocation density as a function of aging time.

Figure 4a illustrates the size of the subgrain with increasing aging time from 245 nm to 475 nm for aging up to 30,000 h. Panait [33,34] investigated the microstructural evolution in 9% Cr heat-resistant steel during long-term creep and thermal aging and found that the subgrain size increased significantly under creep stress conditions and only slightly increased under unstressed thermal aging conditions. The results of the present study are consistent with the above findings and provide good evidence that the subgrain structure is very stable under unstressed thermal aging conditions. Maruyama et al. [13] discovered that the M23C6 carbides are much more effective in controlling subgrain coarsening than the MX carbide, which was attributed to effective pinning of the subgrain boundaries provided by the higher volume fraction M23C6 carbides.

Combining the time-dependent curves of M23C6 carbide size in Figure 3a, it can be concluded that the increasing rate of the subgrain size is in general consistent with that of the M23C6 carbide size, which indicates the important role of the M23C6 carbide in maintaining the stability of the subgrain boundaries by pinning effect. Therefore, the M23C6 carbide must also be effectively maintained within a certain size range in order to stabilize the subgrain boundaries.

There are a high density of dislocations and substructures such as dislocation cells and dislocation walls in the matrix after standard heat treatment [35], which can be clearly shown in Figure 1b. A series of changes will occur in the dislocation density with increasing aging time. Therefore, it is necessary to measure the high-density dislocations in heat-resistant steel by XRD. The dislocation density is measured by the modified Williamson–Hall (MWH) equation through the changes of the half-width of the diffraction peak, which has been widely used in the measurement of dislocation density in heat-resistant steels such as 10Cr-5W and 12% Cr rotor steels [35,36,37]. The modified Williamson–Hall equation can be expressed by the Formula (2):

(2) $$\Delta K\;≅\;\frac{1}{d}+(\frac{{\pi M}^{2}b^{2}}{2}\rho^{1/2}{(K}^{2}{\bar{C})+O(K}^{4}\bar{C})$$

where *K* and Δ*K* represent diffraction vector intensity and integrated width of diffraction peak, respectively; *K* = 2sin*θ*/λ, Δ*K* = 2cos*θΔθ*/*λ*, *θ*, and Δ*θ* are the diffraction angle and the integral width of the diffraction peak; and λ is the X-ray wavelength, 0.15405 nm. *d*, ρ and b are average grain size, the dislocation density, and the Burger vector (0.253 nm), respectively. The value of *M* is determined by the effective outer cut-off radius of dislocations and the dislocation density of the material, according to the existing research [38,39]. *M* = 1; $\bar{C}$ represents the average contrast factor of dislocations for a particular reflection, which can be expressed as Equation (3):

(3) $${\bar{C}=\bar{C}}_{h00}[1-q(\frac{h^{2}k^{2}+h^{2}l^{2}+k^{2}l^{2}}{{\left(h^{2}+k^{2}+l^{2}\right)}^{2}})]$$

where ${\bar{C}}_{h00}$ is the average contrast factor corresponding to the *h*00 reflection; the value of ${\bar{C}}_{h00}$ is considered to be~0.332 ± 0.015 [40], and *q* is a constant which is related to the crystal structure and dislocation type of the test material according to previous research results.

The value of *q* can be obtained when the measured Δ*K* value is closest to the Δ*K* value obtained by the linear fitting calculation of Equation (3), and *q* = 2.60 is obtained by applying MATLAB software to calculate the linear fitting process. The modified Williamson–Hall equation was obtained by linear fitting to the Δ*K* and *K*$\bar{C}$^1/2^ coordinates for each (*hkl*) shown in Figure 4b. The slope (*m*) obtained in Figure 4b can be substituted into Equation (4) to calculate the dislocation density *ρ*:

(4) $$\rho =2\frac{m^{2}}{{\pi M}^{2}b^{2}}$$

After the above analysis, the evolution of the dislocation density for the test steel at 650 °C with different thermal aging times is shown in Figure 4c. The dislocation density in the initial state was as high as 3.2 × 10^14^ m^−2^, and subsequently decreased significantly. When the aging time was 8000 h, the dislocation density decreased to 9.6 × 10^13^ m^−2^. Furthermore, the dislocation density decreased at a relatively slower rate.

The main reason is that the long-term thermal aging process provides the possibility for the cancellation of opposite sign dislocations and the formation of dislocation cells or dislocation walls until the multilateralization process. The coarsening of the subgrain leads to a decrease in the free dislocation density, so the evolution of the subgrain size and dislocation density exhibits opposite characteristics, which is clearly demonstrated in Figure 4a,c. The evolution process of dislocation density is mainly divided into two stages according to Figure 4c: the rapidly decreasing stage and the slowly decreasing stage. The high-temperature environment provides the possibility for the slip and climb of dislocations. When the temperature remains unchanged, the promotion effect of the activation energy provided by the high temperature on the movement of dislocations can be considered to be constant. However, the evolution of the precipitated phases affected by long-term thermal aging showed obvious differences, which was illustrated in Figure 3. When the time aging is short, the content and size of the precipitated phases are at a low level, which make it difficult to effectively hinder the movement of dislocations. Therefore, it is manifested that the dislocation density decreases significantly during short-term aging. When the aging time is extended to 8000 h, a large amount of M23C6 carbides and MX carbonitrides occurred at the martensite lath boundary or subgrain boundary. The effect on hindering the movement of dislocations is more obvious with the increase in the content and size of the precipitated phases, which is not conducive to the recovery of free dislocations, resulting in a slower decrease in the dislocation density with further extension of the aging time.

### 3.4. Creep Behaviors after Different Aging Conditions

**Figure 5:**
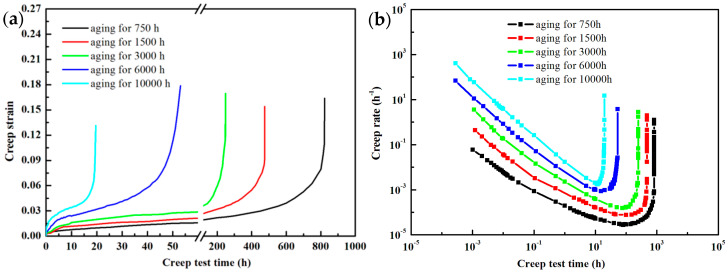
The variation of creep strain and creep rate at 650 °C/160 MPa with different aging times: (**a**) creep strain for aging time; and (**b**) curves of creep rate as a function of time on a double logarithmic scale.

On the basis of the variation characteristics of creep rate with time, the creep deformation process can be divided into three stages in the creep curve [41]. The first stage is the decelerating creep stage, where the creep rate gradually decreases with increasing creep strain, and the deformation hardening effect caused by dislocation proliferation and dislocation interaction at this stage will contribute to the increase in rheological stress. Subsequently, the second stage is the steady-state creep stage, in which the creep rate tends to be constant over a certain strain range, and process hardening and reversion softening by way of dislocation annihilation and rearrangement reach a dynamic equilibrium, where the creep rate is described as the minimum creep rate or steady-state creep rate. Eventually, the third stage is the accelerated creep stage. The creep rate increases dramatically with increasing strain levels, while cracks and holes appear within the material leading to eventual fracture [42,43], which is consistent with the experimental results from Figure 5 in this paper. When there are significant differences in the microstructure within the material, the creep curve still maintains the characteristics of the three stages, but the proportion of each stage can be significantly different.

Figure 5a illustrates the variation of creep strain at 650 °C/160 MPa with different aging times. Furthermore, creep fracture life gradually decreases from 823 h to 19 h for aging up to 10,000 h. Figure 5b shows the creep strain rate versus time on a double logarithmic scale, and the characteristics of the creep rate over time are consistent with the three stages of the creep process described above. Moreover, it can be observed that the minimum strain rate gradually increases as the thermal aging time continues, which indicates some degradation in creep strength [44], which is explained by the coarsening of the subgrain and the instability of the precipitates during the aging process [34,45]. During the short aging time (<1500 h), the precipitates have a beneficial effect on the creep strength, and the dramatic decrease in dislocation density and the rapid increase in subgrain size during this period play a major role in the creep strength [46], resulting in a significant decrease in creep fracture life. The reduction in creep strength of heat-resistant steels during long-term aging can be attributed to the coarsening of subgrains and the rapid coarsening of precipitates, especially the Laves phase and M23C6.

There is still a divergence of opinions about the influence of the Laves phase on the creep performance of martensitic heat-resistant steels. Some believe [23] that the formation and growth of the Laves phase consume the Mo and W elements in the martensitic matrix, and for this reason, their solid solution strengthening effects are effectively reduced, which has a negative effect on creep properties. While others believe [24] that the role of the Laves phase is similar to that of M23C6 without significant coarsening, which can effectively impede the migration of sub-grain boundaries and, therefore, has a positive effect on improving creep strength. However, it is detrimental to creep properties when the size of the laves phase increases significantly [47].

The morphology, size, and chemical composition of M23C6 carbides undergo significant changes during long-term aging. The short rod-shaped M23C6 gradually spheroids and coarsens, while the Cr element continues to enrich around the carbide, which weakens the solid solution strengthening effect by absorbing the Cr elements in the matrix.

The coarsening kinetic behavior of the precipitates in Figure 3 shows that the coarsening of M23C6 weakens the pinning effect on the subgrain boundaries and leads to the significant coarsening of the subgrain, which is the main reason for the significant reduction in creep properties of the material after long-term aging.

Although the MX phase is also relatively effective in hindering the migration of subgrain boundaries during long-term aging, however, it is considered that the effect of the MX phase on the degradation of creep properties can be disregarded due to its low volume fraction and high stability.

**Figure 6:**
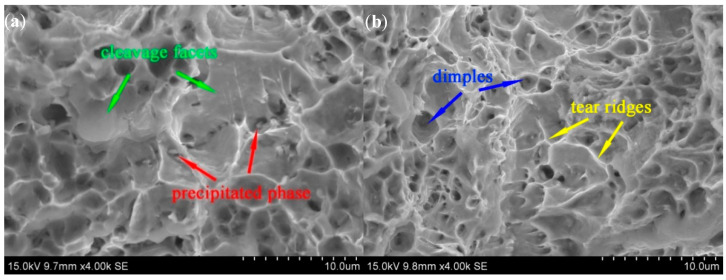
Fracture microscopy of thermal aging creep specimens (6000 h): (**a**) cleavage facets and precipitated phase at the base of dimples; and (**b**) tear ridges and dimples.

The fracture morphology of a creep fracture specimen aged for 6000 h is illustrated in Figure 6, which consists of small dimples, large cleavage facets, and a few tear ridges. Moreover, there is an appearance of more granular precipitated phases at the base of the dimples. The presence of more granular precipitates at the base of the dimples indicates that the Laves phase and M23C6 precipitates during aging and creep deformation are the main sites of crack nucleation, especially when the aging time is extended further; the influence of the volume fraction and size of the precipitates on the creep fracture life becomes more apparent.

## 4. Conclusions

The microstructural evolution under different conditions and its effect on creep behavior of 11Cr-3W-3Co martensitic ferritic steel at 650 °C for up to 30,000 h have been investigated by means of SEM, EDS, TEM, and XRD in the present study. The major results obtained are summarized as follows:

1. Laves phase formed in 11Cr-3W-3Co steel after short-term aging (>750 h) at 650 °C and grew obviously once formed. Furthermore, some Laves phases nucleated at the interface of M23C6 particles. Higher contents of Si and P are good promoters of the nucleation of the Laves phase during long-term aging.
2. The coarsening behavior of different precipitated phases as well as the subgrain was further investigated; the Laves phase and M23C6 carbides continued to grow at a certain rate with increasing aging time. The Laves phase has a rapid increase from 176 nm to 943 nm and the size of M23C6 starts from 79 nm to 272 nm. MX showed a fairly stable size from 53 nm to 81 nm as the aging time is extended to 30,000 h. The subgrain size increased from 245 nm to 475 nm for aging up to 30,000 h. The rate of increase in subgrain size is, in general, consistent with that of the M23C6 carbide size. The evolution of the dislocation density in different aging times shows an obvious difference, and the decreasing rate of dislocation density is significantly affected by the precipitated phase after long-term aging time.
3. Furthermore, the creep behavior at 650 °C/160 MPa under different aging conditions was discussed. The creep performance of the material decreases significantly as the aging time increases and the minimum strain rate gradually increases as the thermal aging time continues, which is closely related to the coarsening of the precipitates, especially M23C6 and the subgrain during long-term aging.

## Figures and Tables

**Table 1:** The chemical composition (wt.%) of the 11Cr-3W-3Co steel.

Element	C	Cr	W	Co	Si	Mn	Ni	Mo	V	Nb	N	B	Fe
wt.%	0.10	10.7	2.7	2.9	0.1	0.5	0.6	0.25	0.19	0.08	0.02	0.016	Bal.

**Table 2:** The parameters of long-term thermal aging process at 650 °C.

Aging temperature (°C)	650										
Aging time (h)	750	1500	3000	6000	8000	10,000	15,000	18,000	21,000	25,000	30,000

## Data Availability

The datasets generated during and/or analyzed during the current study are available from the corresponding author on reasonable request.
